# Recent Advances of Osterix Transcription Factor in Osteoblast Differentiation and Bone Formation

**DOI:** 10.3389/fcell.2020.601224

**Published:** 2020-12-15

**Authors:** Qian Liu, Mao Li, Shiyi Wang, Zhousheng Xiao, Yuanyuan Xiong, Guangwei Wang

**Affiliations:** ^1^Key Laboratory of Brain and Neuroendocrine Diseases, College of Hunan Province, Hunan University of Medicine, Huaihua, China; ^2^Biomedical Research Center, Hunan University of Medicine, Huaihua, China; ^3^XiangYa School of Medicine, Central South University, Changsha, China; ^4^Department of Medicine, University of Tennessee Health Science Center, Memphis, TN, United States; ^5^Department of Neurosurgery, The Second Affiliated Hospital of Nanchang University, Nanchang, China

**Keywords:** osterix, osteoblast differentiation, therapy, osteolytic diseases, bone

## Abstract

With increasing life expectations, more and more patients suffer from fractures either induced by intensive sports or other bone-related diseases. The balance between osteoblast-mediated bone formation and osteoclast-mediated bone resorption is the basis for maintaining bone health. Osterix (Osx) has long been known to be an essential transcription factor for the osteoblast differentiation and bone mineralization. Emerging evidence suggests that Osx not only plays an important role in intramembranous bone formation, but also affects endochondral ossification by participating in the terminal cartilage differentiation. Given its essentiality in skeletal development and bone formation, Osx has become a new research hotspot in recent years. In this review, we focus on the progress of Osx’s function and its regulation in osteoblast differentiation and bone mass. And the potential role of Osx in developing new therapeutic strategies for osteolytic diseases was discussed.

## Introduction

Nearly two decades ago, osterix (Osx) was first discovered by Nakashima et al. (2002). Osx, also known as Sp7, is a zinc finger-containing osteoblast-specific transcription factor belonging to the SP/KLF family (Nakashima et al., 2002; Suske et al., 2005). Its subcellular localization is restricted to the nucleus (Nakashima et al., 2002). Osx is expressed in osteoblast-lineage cells, chondrocytes and also overexpressed in various cancer tissues (Qu et al., 2019). The Osx protein is highly conserved between human and mouse with an overall amino acid sequence identity of 95%. The transcription factor Osx induces the expression of a slew of mature osteoblast genes such as collagen type-I a1 (Col1a1), Osteonectin, Osteopontin, Osteocalcin, and Bone sialoprotein (Bsp) which are all necessary for productive osteoblasts during the creation of ossified bone (Renn and Winkler, 2009). In humans, several genome-wide association studies have demonstrated a correlation between Osx’s certain polymorphisms and decreased bone mineral density in children and adults, and clinical researches revealed that Osx is associated with age-related osteoporosis (Calabrese et al., 2017; Kemp et al., 2017; Qaseem et al., 2017). This review aims to discuss the role of Osx in bone formation and bone mass control, signaling pathway network of Osx regulation, as well as Osx potential value in developing new therapeutic strategies for osteolytic diseases.

## The Structure of Osx

**FIGURE 1:**
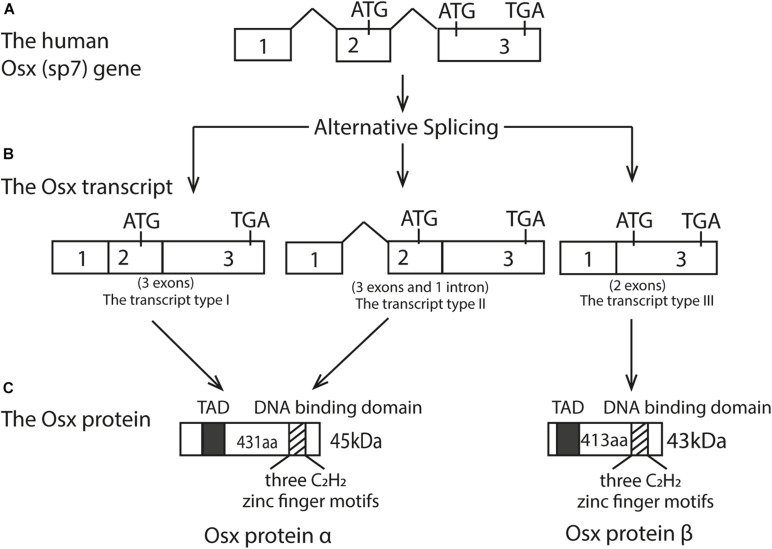
Structure of the Osx gene, its three transcripts, and two protein isoforms. **(A)** Human Osx (sp7) gene; **(B)** The Osx transcripts; **(C)** The Osx protein isoforms. Open boxes represent exons and the thick line represents introns. Three alternatively spliced mRNA isoform were obtained. Because of the absence of initiation codon in the exonl and the same coding sequence between the transcript type I and type II, they eventually translated into the identical protein products. The transcript type III translated into a short protein isoforms with 413 aa. These two protein isoforms differ by the absence or presence of exon2. Therefore, Osx protein B lacks the first 18 N-terminal amino acids compared to Osx protein α. The black box represents N-terminal transcriptional activation domain (TAD) rich in proline and serine. The striped box represents the DNA binding domain containing three C2H2 zinc finger motifs near the C-terminal.

Human Osx is located at chromosome 12q13.13 while in mice, it’s located in chromosome 15q (Nakashima et al., 2002). The initial research found that Osx gene consists of three exons and two introns, with exon2 contains the 5′UTR and encodes a small part of amino acids, while exon3 contains the 3′UTR and encodes most of the protein (Nakashima et al., 2002; Gao et al., 2004). The Osx gene has a TATA-less promoter, and Osx regulates its own promoter through a tandem repeat CCACCC element in its proximal promoter (Barbuto and Mitchell, 2013). The Osx mRNA transcript is an approximately 3.2 kb sequence and three alternatively spliced mRNA variants have been identified with 5′RACE experiments (Gao et al., 2004). Because of the absence of initiation codon in the exon1 and the same coding sequence between the transcript type I and type II, they eventually translated into the identical protein products (Gao et al., 2004). Therefore, the Osx protein can exist as either the long isoform α with 431 residues, derived from the transcript type I and II, or a short isoform β with 413 residues, derived from the transcript type III. As a result, protein β lacks the first 18 N-terminal amino acids compared to protein α, and both isoforms can be visualized on an immunoblot as bands at approximately 45 and 43 kDa, respectively (Milona et al., 2003; Gao et al., 2004; Ramazzotti et al., 2019). The difference between these two protein isoforms is the absence or presence of exon2. In addition, the amino acid sequence of the protein translated by Osx transcription factor was as high as 95%, in which a transcriptional activation domain (TAD) rich in proline and serine at N-terminal (Gao et al., 2004). Osx protein is a sequence-specific DNA binding protein. Its DNA-binding domain is located at the C-terminus and contains three C2H2-type zinc finger domains, which binds to SP1 and EKLF consensus sequences and to other G/C-rich sequences in the target genes (Nakashima et al., 2002). The schematic diagram of the Osx gene genome structure, mRNA transcript and its protein isoforms was shown in Figure 1.

## The Function of Osx in Controlling Bone Formation

**FIGURE 2:**
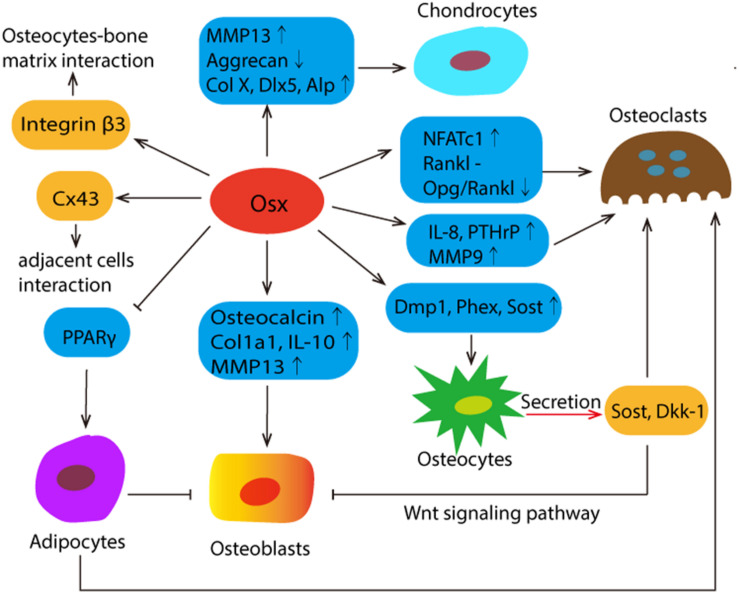
The schematic summary of Osx’s mission in the complex communications among different bone cells and its potential role in bone micro-environment. Osx affects the differentiation, maturation and function by regulating the different target genes, which were presented in the blue box. Osx directly controls the expression of integrin β3, which plays a mediating role in osteocyte-bone matrix interaction. The most abundant gap junction protein Connexin43 (Cx43) was significantly regulated by Osx positively. Osx decreases osteoblast activity by stimulating the expression of sclerostin (Sost) and Wnt signaling pathway inhibitor Dickkopf-related protein 1 (Dkk1). Both of them are predominantly expressed in osteocytes. And Sost also functions as a secreted osteoclast-derived BMP antagonist to promotes osteoclast differentiation. In addition, Osx represses adipogenesis by negatively regulating PPARy expression and transcriptional activity, and adipocytes suppress osteoblasts and promote bone resorption by the recruitment of osteoclasts.

Up to now, it has been shown that Osx is specifically expressed in osteoblasts and osteocytes and, albeit at lower levels, in prehypertrophic and hypertrophic chondrocytes, while not expressed in osteoclasts (Xing et al., 2019). Osx not only plays a vital role in the differentiation, maturation or function of bone cells through the regulation of different genes, but also shows the potential role in the bone micro-environment. The schematic diagrams were summarized in Figure 2.

### The Function of Osx in Osteoblasts

Osx has been confirmed to be involved in osteoblast differentiation, maturation and activity (Ramazzotti et al., 2019). It was reported that osteoblast differentiation does not occur at all in Osx-null embryos (Fu et al., 2010; Zhou et al., 2010). These studies demonstrated that Osx is essential for embryonic skeletal development. In Osx-deficient mice, the differentiation of runt-related transcription factor 2 (Runx2) expressing precursor cells was arrested and unable to express osteoblast markers (Zhou et al., 2010). When the vector expressing Osx was transfected into C2C12 and C3H10T1/2 cells, Osteocalcin RNA was obviously induced by Osx in these two cell types and Col1a1 gene expression was activated in C2C12 cells (Nakashima et al., 2002). Inactivation of Osx mice by CAG-CreER postnatally resulted in an arrest of osteoblast differentiation and of new bone formation, revealing that Osx also plays an indispensable functional role in postnatal skeletal growth and homeostasis (Zhou et al., 2010). An Osx mutation in zebrafish or medaka, belonging to non-mammals, resulted in a general delay in osteoblast maturation or severe bone defects and larval lethality (Azetsu et al., 2017; Niu et al., 2017; Yu et al., 2017), which established a key role of Osx for bone formation in non-mammalian species.

### The Function of Osx in Osteocytes

Osx is necessary for the maturation and function of osteocytes postnatally (Baek and Kim, 2011; Klein-Nulend et al., 2013). Osx postnatal mutants appeared morphological osteocyte abnormalities, unlike normal osteocytes, and the expression levels of proteins encoded by “mineralization-related genes,” such as Dmp1, Phex, and Sost, were significantly reduced (Zhou et al., 2010). The number of osteocytes close to both periosteum and endosteum was decreased and osteocytes were also markedly deformed. The mineralization process was seriously compromised in the Osx postnatal mutants. In both EMSA experiments and intact cells, Osx interacted with a specific site in the sclerostin promoter and activated this promoter in transfection assays, suggesting that Osx is also a player in mature osteocytes (Zhou et al., 2010).

### The Function of Osx in Chondrocytes

The role of Osx in chondrocytes was first reported by Omoteyama and Takagi (2010). They investigated the *in vitro* effects of Osx gene silencing in the chondrogenic cell line ATDC5. Osx’s shRNA down-regulated the expression of type X collagen (Col X), distal-less homeobox 5 (Dlx5) and alkaline phosphatase (Alp) mRNA, attenuated Alp enzyme activity, which suggests that Osx is involved in chondrogenic gene activation and chondrocyte differentiation. As for the *in vivo* effects, in Osx null mutants, there is no abnormality in the cellular organization of the cartilage growth plate (Nakashima et al., 2002). However, endochondral ossification completely stopped at the hypertrophic stage in chondrocyte-specific Osx conditional KO mice, even resulting in postnatal lethality combined with respiratory insufficiency (Omoteyama and Takagi, 2010; Oh et al., 2012). Massive accumulation of calcified cartilage and diminishment of bone trabecula were observed in Osx-floxed mice with the Col2a1-Cre-ERT2 transgene, which was caused by a delay in the development of hypertrophic chondrocytes and their conversion to osteoblasts (Zhou et al., 2010; Xing et al., 2019).

### The Function of Osx in Osteoclasts

Although Osx is not expressed in osteoclasts, a number of scientific studies have suggested that Osx has different effects on osteoclasts. Receptor activator of NF-kappaB ligand (Rankl) signaling is the major determinant of osteoclast formation and activation, while osteoprotegerin (Opg) protects bone from excessive resorption by binding to Rankl. Their relative concentration is of great significance for bone mass and strength. A research conducted by Cao et al. (2005) found that the decrease of osteolysis was followed by Osx gene transfection, but the transfection of Osx did not inhibit Rankl expression. However, Zhou et al. (2010) have shown that the ratio of Opg/Rankl expression in long bones was increased in the Osx postnatal mutants. Consequently, the fewer overall number and size of osteoclasts was observed in Osx postnatal-null long bones. This contradicted result declared that more studies about the mechanisms of Osx’s inhibiting effect on osteoclasts are necessary. Cytokines like interleukin-8 (IL-8) and parathyroid hormone-related protein (PTHrP) can cause bone destruction by inducing osteoclast differentiation and activation, they were also increased by Osx over-expression (Yao et al., 2019). An essential transcriptional factor for osteoclast differentiation named nuclear factor of activated T cells 1 (NFATc1) was also studied. It forms a complex with Osx and activates Osx-dependent Col1a 1 promoter, suggesting that Osx has different effects on osteoclasts (Koga et al., 2005; Canalis et al., 2020).

### The Promising Role of Osx in the Complex Communications Among Different Bone Cells and Its Role in the Bone Micro-Environment

The achievement and maintenance of a healthy and stable bone mass is accomplished through a close crosstalk among bone cells. Increasing evidences have shown that Osx not only performs multiple functions in different bone cells, but also participates in the cross-talk among them. Sclerostin (Sost) and Dickkopf-related protein 1 (Dkk1) are predominantly expressed in osteocytes, they exhibit a suppressive effect on osteoblast activity and function by antagonizing the Wingless-type and Int (Wnt) signaling pathway. Osx decreases osteoblast activity by stimulating the expression of Sost and Dkk1. As a secreted osteoclast-derived BMP antagonist, Sost not only inhibits osteoblast differentiation but also promotes osteoclast differentiation (Kusu et al., 2003). In addition to this, Osx can activate the Sost promoter and specifically bind to a DNA fragment located within the promoter (Cao et al., 2015; Zhang Z. H. et al., 2020). The osteocytes are surrounded by a non-organized pericellular matrix and integrins play a mediating role in osteocyte-bone matrix interaction. Osx directly controls the expression of integrin β3, which regulates corticalization for longitudinal bone growth (Moon et al., 2018). Osteoblasts and osteoclasts can communicate with each other. Osteoclasts produce factors called clastokines controlling osteoblasts during the bone remodeling cycle. Matrix metalloproteinase-9 (MMP9), predominantly produced by osteoclasts in bone, have an important function at the injured bone absorption, healing and bone remodeling after dental implant placement. It was reported that MMP9 initiates osteoclasts by removing collagen from the demineralized bone (Liu et al., 2004; Chen et al., 2018; Zhang H. et al., 2020). MMP9 is the target of Osx, in which Osx is recruited to the MMP9 promoter and binds to the CCAAT regulatory element of MMP9 promoter. Correspondingly, it has been clearly demonstrated that osteoblasts also affect the activity of osteoclasts, and Osx participates in the cross-talk between them by up-regulating factors expressed in osteoblasts such as Matrix metalloproteinase-13 (MMP13) (Hayami et al., 2011; Nakatani et al., 2016). MMP13 is expressed in hypertrophic chondrocyte and osteoblast. It contributes significantly to differentiation of osteoblast. Meanwhile, MMP13 also plays a significant role in differentiation and activation of osteoclast (Hayami et al., 2011; Pivetta et al., 2011; Nakatani et al., 2016). In osteoblasts, Osx activates the MMP13 promoter activity in a dose-dependent manner (Zhang et al., 2012). These studies suggested that Osx is involved in the interaction of osteoblast and osteoclast by mediating different factors. As the most abundant gap junction protein in bone cells, Connexin43 (Cx43) participates in the communication between adjacent cells, as well as cells and extracellular environment (Chen et al., 2019). It was reported that Cx43 expression was significantly repressed by the addition of shRNA against Osx, whereas overexpression of Osx resulted in enhanced Cx43 expression. Further studies have proven that Osx can directly occupy the promoter region of Cx43 and subsequently increases Cx43 promoter activity in a dose-dependent manner (Han et al., 2016a; Chen et al., 2019). Recent studies have shown that bone marrow adipocytes not only suppress osteoblasts, but also promote bone resorption by the recruitment of osteoclasts, and Osx represses adipogenesis by negatively regulating PPARγ expression and transcriptional activity (Han et al., 2016b; Li et al., 2018b).

## Osx Promotes Osteogenesis Through the Regulation of Downstream Factors

The essential role of Osx in osteoblast differentiation is attributed to its ability to regulate the expression of various osteoblast markers such as Bsp, fibromodulin, Osteocalcin, Dkk1 and Col1a1, etc. (Wu et al., 2007; Ortuno et al., 2013; Yang et al., 2016; Niu et al., 2017). In addition to these target genes previously discovered, several novel downstream targets of Osx have been identified. Zinc finger and BTB domain containing 16 (Zbtb16), a downstream target gene of Osx, functions as a late marker of osteoblastic differentiation and regulates osteogenesis of human multipotent mesenchymal stromal cells (Onizuka et al., 2016). Fibrillin-2 and periostin are also identified to be target candidates of Osx in osteoblast differentiation (Lee et al., 2017). Besides, Osx regulates corticalization by controlling integrin β3 expression directly (Moon et al., 2018). Osx increases the promoter activity of Cx43 by directly interacting with the Cx43 promoter and subsequently upregulates the expression level of Cx43. As a result, the expression and transcriptional activity of Cx43 were considerably affected by Osx (Han et al., 2016a).

It is generally recognized that the canonical Osx pathway usually involves binding to GC-box DNA elements to regulate the transcription of target genes. Osx is able to activate Bsp promoter reporter in a dose-dependent manner, and one GC-rich site is required for Bsp promoter activation by Osx directly (Yang et al., 2016). Contrary to expectation, it has been reported that Osx acts as a transcriptional co-activator in the distal-less homeobox (Dlx) regulatory complex that binds to AT-rich motifs (Hojo et al., 2016). It’s obvious from this point that Osx can also form complexes with other transcription factors to co-regulate downstream target genes. Osx can also interact with Runx2 to coordinately activate the expression of the various genes, and their synergistic effects achieve significantly higher expression levels than those obtained with the individual expression vectors. Col1a1, Sost, Ectonucleotide pyrophosphatase/phosphodiesterase 1(Enpp1) and the novel gene unique cartilage matrix-associated protein (Ucma) have been reported to be their coordinated target genes (Ortuno et al., 2013; Lee Y. J. et al., 2015; Pérez-Campo et al., 2016; Gao M. et al., 2018). The interaction of Osx and Runx2 in the regulation of these promoters is mediated by Osx’s enhancer regions adjacent to Sp1 and Runx2 DNA-binding sites, thereby synergistically regulating those downstream genes transcription.

More notably, both Runx2 and Osx are induced by Zn^2+^ influx, and they transcriptionally regulated ZIP1 expression which further leads to induction of Zn^2+^ influx contributing to a positive feed-forward zinc-Runx2/Osx-ZIP1 regulation loop during osteogenic differentiation (Karieb and Fox, 2012; Fu et al., 2018). NFATc1 forms a complex with Osx and activates Osx-dependent Col1a 1 promoter, however it does not activate Runx2-dependent transcription. Furthermore, transcriptional regulators such as p300, Brg1 or NO66 have been shown to interact with Osx and regulate its transcriptional activity (Ortuño et al., 2010; Sinha et al., 2014). It is noteworthy that Osx and NO66 histone demethylase control the chromatin of its target genes, in which Osx acts as a molecular switch for the formation of an active chromatin state during osteoblast differentiation, whereas NO66 suppresses gene through histone demethylation and/or formation of a repressor complex. Osx and NO66 work together to achieve multi-layered control of the chromatin structure of target genes (Sinha et al., 2010; Sinha et al., 2014).

The investigation of downstream targets of Osx contributes to elucidate its molecular mechanism affecting osteoblast differentiation and bone formation, thereby further promoting the exploration of new regulatory mechanisms involving Osx. The Osx direct and indirect downstream targeting molecules for osteoblastic differentiation and their mode of action (MOA) were sumarized in Table 1.

**TABLE 1:** The Osx direct and indirect downstream targeting molecules for osteoblastic differentiation and their mode of action (MOA).

**Target genes**	**MOA of Osx**	**Functions**	**Reference(s)**
Bsp	By binding to specific GC-rich site directly	Up-regulation	Yang et al., 2016
Fibromodulin	By binding to specific GC-rich site directly	Up-regulation	Ortuño et al., 2010
Sost	By binding to specific GC-rich site directly and activating Sost expression with Runx2 in a co-ordinated manner	Up-regulation	Yang et al., 2010; Pérez-Campo et al., 2016
Periostin	By binding to specific GC-rich site directly	Up-regulation	Lee et al., 2017
Osteocalcin	By binding to the CCAAT sequence directly	Up-regulation	Niger et al., 2011
Dkk1	By enhancing Dkk1 expression directly	Up-regulation	Cao et al., 2015
Col1a1	By binding the Sp1 boxes directly or forming a complex with NFATc1 to upregulate the Col1a 1 expression indirectly	Up-regulation	Koga et al., 2005; Ortuno et al., 2013
Col1a2	By binding to the second GC-rich site directly	Up-regulation	Yano et al., 2014
Col5a1	By binding to GC-rich sequence directly	Up-regulation	Wu et al., 2010a,b
Col5a3	By binding to GC-rich sequence directly	Up-regulation	Wu et al., 2010a,b
Zbtb16	By binding to the SP1-binding site directly	Up-regulation	Onizuka et al., 2016
Integrin β3	By binding to the integrin β3 promoter directly	Up-regulation	Moon et al., 2018
Cx43	By binding to the promoter region of Cx43 directly	Up-regulation	Han et al., 2016a
Vascular endothelial growth factor (VEGF)	By binding to the promoter directly or regulating gene expression of VEGF with HIF-1α cooperatively	Up-regulation	Chen et al., 2012; Tang et al., 2012
MMP13	By binding to the GC-rich sequence directly	Up-regulation	Zhang et al., 2012
MMP9	By binding to the CCAAT sequence directly	Up-regulation	Yao et al., 2019
ZIP1	By regulating gene ZIP1 expression of VEGF with Runx2 cooperatively	Up-regulation	Fu et al., 2018
Ucma	By binding to Sp1-binding sites directly	Up-regulation	Lee Y. J. et al., 2015
Enpp1	By binding to Sp1-binding sites directly	Up-regulation	Gao M. et al., 2018
Fibrillin-2	Unknown	Down-regulation	Lee et al., 2017

## The Signaling Pathways Control Osx Expression

**FIGURE 3:**
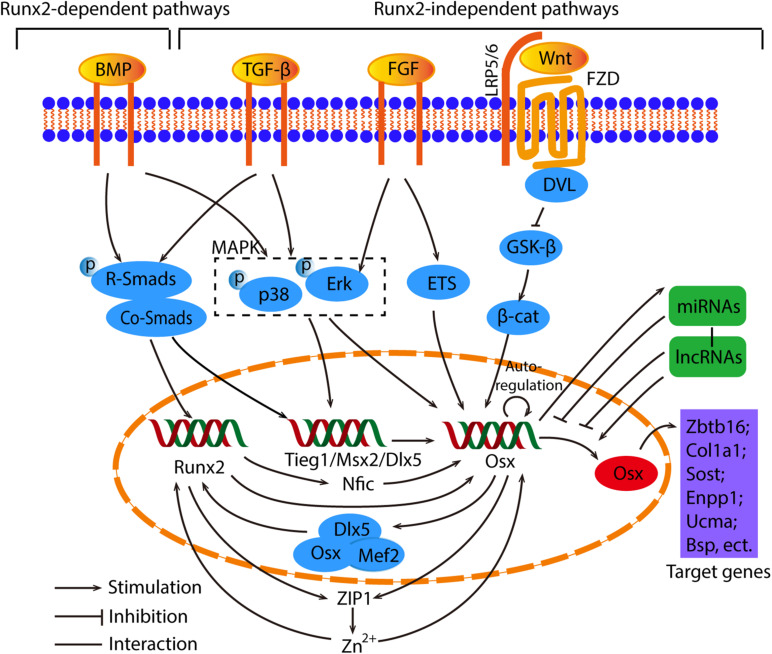
The overview of crosstalk pathways associated with Osx. The signaling pathways that control Osx expression can be divided into Runx2-dependent and Runx2-independent pathways. In regard to the Runx2 dependent pathways, the BMP/Smads/Runx2 is the canonical pathway, and Runx2 regulates Osx by Nfic, ZIP1 indirectly or upregulate Osx directly. Meanwhile, Osx cooperate with Dlx5 and Mef2 to control Runx2 reversely. As for the Runx2-independent pathway, they are induced by BMP, TGF-β and FGF, then activate Smads, MAPK or ETS to regulate Osx directly or indirectly. The Wnt/β-catenin signaling is involved in the suppression of Osx. What’s more, Osx has the capability of auto-regulation. Osx are also controlled by miRNAs and lncRNAs in an epigenetic manner. Generally, miRNAs act as negative regulators and Osx conversely stimulates miRNAs. Different lncRNAs regulate Osx positively or negatively. Eventually, Osx promotes osteogenesis through the regulation of downstream factors such as Zbtb16, Col1a1, Sost, etc.

There are two main pathways which cause in the induction of Osx gene expression, indirectly or directly (Ramazzotti et al., 2019). We will discuss these two signaling pathways in detail below. The overview of crosstalk pathways associated with Osx were showed in Figure 3.

### Runx2-Dependent Pathways

It has been extensively recognized that Osx functions as downstream of Runx2, since the latter is expressed in mesenchymal cells of Osx KO mice while Osx expression is not observed in the absence of Runx2 (Komori, 2018). For the Runx2-dependent pathway, it is usually activated by bone morphogenic protein (BMP) signals (Li et al., 2017, 2018a; Lee et al., 2019). Interestingly, Osx was also first discovered as a BMP2 induced gene (Lee et al., 2003). BMP2 plays a unique role in mesenchymal stem cells (MSCs) differentiation by controlling the transition from progenitors to Runx^2+^ Osx^+^ cells. Osx expression is not regulated by the orchestration of the BMP signaling pathways directly and specifically but eventually by crucial transcriptional factors (Matsubara et al., 2008). BMP2 is proved to activate Runx2 through Smad signaling and Runx2 in turn up-regulates Osx expression, in which Runx2 directly binds to Osx promoter region and regulates Osx promoter activity (Xiao et al., 2004; Pérez-Campo et al., 2016; Takarada et al., 2016). Nuclear factor I-C (Nfic), expressed in human osteoblasts and osteoblast-like cell lines, was found to be a new candidate gene that participate in osteogenic differentiation. It acts as an intermediary transducer between Runx2 and Osx in the BMP2 signaling pathway where Runx2 is upstream of Nfic and Nfic directly controls Osx expression (Lee et al., 2014; Zhang et al., 2015; Lee et al., 2018). Osx is localized on the enhancer region in primary osteoblasts, and can form an enhanceosome with Dlx5 and myocyte enhancer factor 2 (Mef2) to synergistically activate an osteoblast-specific enhancer of Runx2, demonstrating that Osx is also involved in the regulation of Runx2 expression (Kawane et al., 2014). These suggest that the regulation between Osx and Runx2 works like a positive loop indirectly.

### Runx2-Independent Pathways

Despite both Runx2 and Osx control bone mineralization and MSCs differentiation, the bone phenotype of Osx-deficient mice differ from that of Runx2-deficient mice (Nakashima et al., 2002; Caparros-Martin et al., 2013), indicating their distinctive functions during the process of bone formation. Osx could even bind and stimulate the upstream CCACCC site in its promoter to regulate its own expression, forming a positive feedback mechanism (Barbuto and Mitchell, 2013). It has been shown that BMP2 and Msh homeobox 2 (Msx2) induced Osx expression in Runx2-deficient mesenchymal cells, and the knockdown of Msx2 blocked the induction of Osx in the Runx2-deficient MSCs, which indicates that BMP2 regulates Osx expression through Msx2 independently of Runx2 (Matsubara et al., 2008). Similarly, a novel factor necessary for optimal expression of Osx in osteoblasts, namely TGFβ-induced early gene 1 (Tieg1), is also required for BMP2 and TGFβ-mediated Osx expression. It directly regulates the expression of Osx by binding to its proximal promoter (Subramaniam et al., 2016; Rajamannan, 2019).

According to reports, MAPK is also involved in the BMP2-induced Osx expression (Sun et al., 2018). BMP2-mediated enhancement of Osx mRNA transcription is achieved through the activation of Dlx5 by p38 and extracellular signal-regulated kinase (Erk)-mediated phosphorylation. During this process, the Dlx5 binds to the Osx promoter and recruits p300, a co-activator, to increase the stability of Osx (Lee et al., 2003; Choi et al., 2011a; Xiao et al., 2015; Abdallah et al., 2018). Based on the studies of the signaling pathways related to MAPK, the mechanism of osteoporosis treatment is being further understood. For example, a significant decreases in the protein levels of Runx2 and Osx under Erk1/2, p38, or c-Jun-N-terminal kinase (JNK) signaling inhibitor treatment in β-tricalcium phosphate (β-TCP)/Mg-Zn composite can be observed easily, indicating that Mg^2+^ in Mg-Zn extract promotes osteogenic differentiation via p38 MAPK-regulated Osx (Wang Z. et al., 2020).

The wingless-related integration site (Wnt) pathway modulates bone formation through the control of progenitor cells proliferation and differentiation (Sun et al., 2016; Jing et al., 2018; Yang et al., 2020). The Wnt1 class activates the canonical Wnt signaling pathway by binding to lipoprotein receptor-related protein 5 and 6 (LRP5/6). And the canonical Wnt signaling pathway increases the stability and accumulation of β-catenin in the cytoplasm, therefore facilitating the entry of β-catenin into the nucleus to promote target genes Osx transcription (Liu et al., 2015; Nemoto et al., 2016; Shi et al., 2016). A process in the developing facial skeleton was investigated and showed that Osx is a transcriptional target of the fibroblast growth factors (FGFs) pathway. Its manipulation has an immediate and strong effect on Osx expression and FGFs directly activate Osx expression via a shared intronic *cis*-regulatory module. The activity of the FGFs pathway was modulated by Wnt/β-Catenin pathway, and the interactions between FGFs and Wnt/β-Catenin signaling pathways were mediated by ETS factors (Felber et al., 2015). Wnt3a upregulates Osx expression through activation of p38 MAPK in dental follicle cells, but p38 MAPK signaling has no crosstalk with phosphorylation of the glycogen synthase kinase-3β (GSK3β) and accumulation or translocation of β-catenin (Sakisaka et al., 2016). Osx can synchronously work with HIF-1α to further inhibit β-catenin activity. Similarly, Osx suppresses the activity of the canonical Wnt signaling pathway in osteoblasts by activating the Wnt antagonist Dkk1 (Cao et al., 2015).

## Other Factors in the Bone Microenvironment Interact With Osx

The secretion of hormones and cytokines in the bone microenvironment has a significant effect on the differentiation of osteoblasts and osteoclasts. And these molecules interact with Osx to influence osteogenic differentiation, which provides new avenues to develop therapeutic strategies for osteolytic diseases.

It is well-known that estrogen (ER) deficiency has clearly been established as seminal mechanism in the pathogenesis of osteoporosis (Farr et al., 2019). The lack of estrogen leads to disorders in the regulation of cytokines, growth factors and humoral factors in the bone microenvironment. And the molecular mechanism for the role of ER in bone cells is being unraveled gradually. Interestingly, it has been certificated that ER exert its function of promoting osteogenic differentiation by elevating Osx expression (Han et al., 2020). Parathyroid hormone (PTH) has been proved to increase the Osx expression levels (Yang et al., 2019). Secretion of melatonin is regulated by the suprachiasmatic nucleus of the hypothalamus. Recently, several findings have demonstrated that melatonin regulates Osx expression through inhibition of the ubiquitin-proteasome system, and therefore increases Osx-mediated Alp activity, matrix mineralization, and transcriptional activity. Furthermore, the occupancy of Osx at the promoter of the Bsp gene is also enhanced by melatonin. Researchers believe that melatonin may be a potent osteogenic agent in the treatment of osteoporosis (Machida et al., 2006; Han et al., 2017; Choi et al., 2018; Zhou et al., 2020). Another hormone neuropeptide Y, produced by osteoblasts and other peripheral tissues, was also proved to directly promote osteogenic differentiation of MC3T3-E1 cells by upregulating Osx *in vitro* (Zhang B. et al., 2020).

The bone is the third most common site of metastasis for a wide range of solid tumors. When metastatic cancer cells invade the bone, the crosstalk between tumor cells and the bone microenvironment disrupts the bone homeostasis. It was reported that galectin-3, a tumor-secreted sugar-binding protein, regulates the expression of Osx, thereby remodeling bone in the bone microenvironment niche (Nakajima et al., 2014). Opn released from cancer stem cells acts as a stimulator of osteogenesis by regulating Osx (Kim et al., 2019). In addition, expressions of two cytokines, interleukin-8 (IL-8) and parathyroid hormone-related protein (PTHrP), that cause bone destruction by inducing osteoclast differentiation and activation, were increased by Osx over-expression. On the contrary, there is low IL-8 and PTHrP expression in the tumors with Osx-knockdown cells (Yao et al., 2019). Interleukin-10 (IL-10), a cytokine that directly increases osteoblast differentiation and inhibits osteoclast differentiation, is able to up-regulate Osx gene expression in osteoblasts via mitogen-activated protein kinase (MAPK) pathway (Rios-Arce et al., 2020). These results demonstrated that Osx has potential regulatory effects on various molecules in the bone microenvironment.

## miRNAs and lncRNAs Regulate Osx Expression

### Osx Is Regulated by lncRNAs in an Epigenetic Manner

Long non-coding RNAs (lncRNAs), a novel subset of non-protein-coding RNAs with longer than 200 nucleotides was established to regulate Osx epigenetically (Wu J. et al., 2018; Wu R. et al., 2018; Jiang et al., 2019; Wang et al., 2019). It was found that the regulation of osteoblast activity by lnc-ob1 is dependent on Osx. lnc-ob1 binds Suz12, a subunit of the Polycomb Repressive Complex 2, to control H3K27me3 methylation at the Osx promoter, thereby effectively regulating the Osx mRNA levels and protein levels (Sun et al., 2019). A recent report illustrated a novel mechanism of Osx during the osteogenic differentiation, that is, lncRNAs regulate Osx expression via a Runx2-independent pathway. Overexpression or knockdown of lncRNA ODIR1(Osteogenic differentiation inhibitory regulator 1) significantly reduced or increased the expression levels of the mRNA and protein level of Osx in the osteogenic differentiation of MSCs while Runx2 was not altered, which strongly indicates that lncRNA ODIR1-mediated Osx expression is not dependent on Runx2. Further research showed that lncRNA ODIR1 inhibits Osx transcription by altering the modification of histone marks on Osx promoter. Increased expression level of F-box protein 25 (FBXO25) by knockdown of lncRNA ODIR1 in human umbilical cord-derived MSCs promoted H2BK120 mono-ubiquitylation which stimulated H3K4 trimethylation, and then the transcription level of Osx was elevated, in which both H2BK120ub and H3K4me3 form a loose chromatin structure and induce Osx expression (He et al., 2019). Consequently, these studies demonstrated that Osx has become a key regulator for researching the mechanism of lncRNAs involved in osteogenic differentiation, and it would be performed to develop novel therapeutic strategies for osteoporosis.

### miRNAs Participate in Epigenetic Regulation of Osx During Osteogenic Differentiation

**FIGURE 4:**
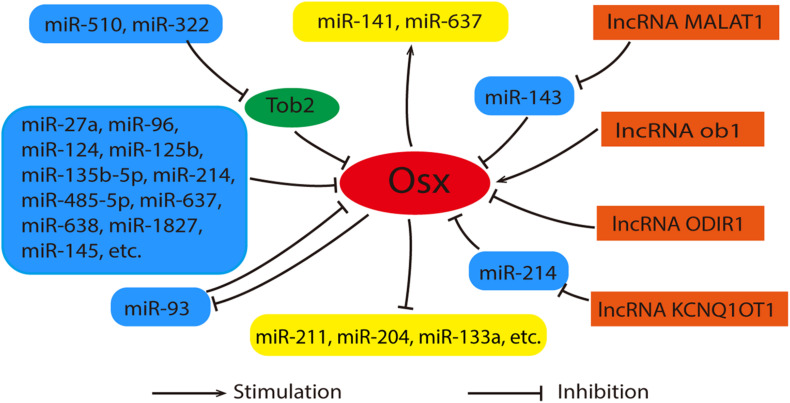
Relationships between Osx, miRNAs and lncRNAs. Generally, miRNAs act as inhibitors during the regulation of Osx. These miRNAs were listed in the left with blue. Among them, miR-322 and rmiR-510 promote Osx expression via the inhibition of Tob2 indirectly. Meanwhile, Osx also has feedback effects on the miRNAs expression positively or negatively. These miRNAs were listed in the middle with yellow. lncRNAs (orange) control the expression of Osx in an direct or indirect manner. As for the indirect way, miRNAs can serve as their intermediary.

As an important post-transcriptional regulator, microRNAs (miRNAs) participate in the osteogenic differentiation of MSCs by targeting multiple genes including Runx2, Dlx, Smad4, etc. (Landgraf et al., 2007; Huang et al., 2016). It has been proved that miR-27a and miR-96 etc. regulate osteogenesis by targeting Osx (As shown in Figure 4) (Zhang et al., 2011; Shi et al., 2013; Zhang et al., 2018; Jiao et al., 2019). They suppress osteogenic differentiation by decreasing Osx expression directly. Recently, Liu et al. (2020) have certified that Osx serves as the direct target of miR-1827 and the inhibitive effect on osteogenic differentiation of miR-1827 amplification was reversed by Osx overexpression. In addition to this, some miRNAs indirectly regulated the expression of Osx via other genes. miR-322 and miR-510 promoted Osx expression via the inhibition of Tob2, a negative regulator of osteogenesis that bound and mediated the degradation of Osx mRNA (Gamez et al., 2013; Wang et al., 2017). Interestingly, a study revealed that Osx also has feedback effects on the miRNAs expression. It was reported that miRNA-93 is able to form a feedback loop with Osx to regulate osteoblast mineralization (Yang et al., 2012). The details were shown in Figure 4.

### lncRNAs Interact With miRNAs to Regulate Osx

Since both miRNAs and lncRNAs have such vital functions in Osx-mediated osteogenic differentiation, is there a possibility that lncRNAs interact with miRNAs to regulate Osx? Some experimental findings correlate with this point of view. In human bone marrow-derived MSCs, lncRNA Metastasis-associated lung adenocarcinoma transcript-1 (MALAT1) is able to increase Osx expression by competitively binding to miR-143 and inhibit its expression, since miR-143 inhibits Osx expression (Gao Y. et al., 2018). Recently, it has been also reported that lncRNA KCNQ1OT1 promotes osteogenic differentiation of bone marrow mesenchymal stem cells (BMSCs) by sponging miR-214 as a competing endogenous RNA via regulating BMP2/Smad pathway (Wang et al., 2019). Obviously, the new molecular mechanism of the interaction between Osx, miRNAs and lncRNAs in regulation of osteogenic differentiation of BMSCs still remains to be explored. For intuitive description, Figure 4 shows the relationships between Osx, miRNAs and lncRNAs clearly.

## Post-translational Modifications Are Important for Osx Activity

Post-translational modification is a key cellular event in controlling the activities of Osx during osteogenic differentiation.

Osteoblast activity is augmented in ubiquitin ligase-deficient mice that generate adult onset osteosclerosis with increased bone mass, indicating that the ubiquitin-proteasome system plays a role in osteoblast differentiation. Generally speaking, proteins are ubiquitinated and then degraded by proteasome. Further studies demonstrated that ubiquitination of Osx plays a key role in osteoblast differentiation. K58 and K230 were first identified to be the ubiquitination sites of Osx by Co-IP assays and protein stability assays (Peng et al., 2013). Subsequently, it has been illustrated that ring finger type E3 ubiquitin ligases Cbl-b and c-Cbl enhance ubiquitin-proteasome-mediated degradation of Osx by reducing the protein stability of Osx in BMP2-stimulated C2C12 cells (Choi et al., 2015). However, Cbl-b and c-Cbl did not affect the protein levels of other osteogenic transcription factors such as Dlx3, Dlx5, and Msx2. These results suggest that Cbl-b and c-Cbl specifically regulate the function of Osx during osteoblast differentiation. CHIP, another novel post-translational regulator of Osx, plays an important role in the regulation of Osx in protein levels in osteoblast precursor cells upon treatment with tumor necrosis factor-α (TNF-α). Unlike the previous description, CHIP mediates the inhibition of Osx by TNF-α in K55 and K386 ubiquitination sites of Osx (Xie and Gu, 2015).

Phosphorylation is widely recognized as an important regulatory pathway in skeletal development and maintenance. Osx can be regulated post-translationally by protein kinase-mediated phosphorylation including NFATc1, protein kinase B (Akt), kinase glycogen synthase kinase-3 (GSK-3), Peptidyl-prolyl isomerase 1 (Pin1) and Erk, etc. in osteoblast differentiation (Koga et al., 2005; Choi et al., 2011b; Qi et al., 2015). Akt is a member of serine/threonine-specific protein kinase that phosphorylates Osx and its activation increases protein stability, osteogenic and transcriptional activity of Osx (You et al., 2011). Mammalian GSK-3, consisting of two isoforms GSK-3α and GSK-3β, plays a vital role in the functional regulation of Osx through phosphorylation modification. Interestingly, different GSK-3 isoforms phosphorylate Osx in a different manner, although the catalytic domains of them exhibit 97% sequence homology. Particularly, GSK-3α phosphorylated Osx at Ser76/80 sites and up-regulated the osteogenic activity of Osx, whereas GSK-3β increased the stability and transactivation activity of Osx through phosphorylation of the newly identified site S422 (Xu et al., 2015). Similarly, the phosphorylation of Ser76/80 of Osx is also important for Pin1 interaction and function (Lee S. H. et al., 2015). What’s more, p38-mediated phosphorylation of Osx at Ser-73/77 enhanced the recruitment of coactivators and then transcriptionally active complexes formed. Further study showed that p38-mediated phosphorylation of Osx increases its interaction with the transcriptional coactivators p300 and Brg1 (Ortuño et al., 2010).

Like phosphorylation, acetylation is a universal modification of proteins by increasing or decreasing the DNA binding and transcriptional activity of transcription factors. CREB binding protein (CBP) co-transfection contributing to Osx acetylation significantly delayed Osx degradation and conversely, histone deacetylase 4 (HDAC4) co-transfection involved in the deacetylation of Osx significantly accelerated Osx degradation. CBP interacts with Osx as a transcription coactivator *in vivo*, resulting in acetylation of the two lysine residues at the C-terminus of Osx. Further studies have shown that Osx acetylation increases its binding to the promoter of the target genes such as Alp, Bsp, Col1a1 and Osteocalcin (Lu et al., 2016). These facts demonstrated that acetylation of Osx enhances its stability and transcription activity. And Osx activity is required for the osteogenic differentiation of C2C12 cells. Therefore, Osx acetylation is necessary to promote osteoblast differentiation. Recently, the effect of Osx acetylation on the osteogenic differentiation of vascular smooth muscle cells (VSMCs) was also studied. After the knockdown of the histone deacetylase Hdac9, the expression of Osx mRNA remained unchanged while the protein expression level was significantly enhanced and then resulted in the enhancement of VSMCs calcification *in vitro*. This process may be mediated by acetylation of Osx (He et al., 2020). It has been confirmed that both endogenous and exogenous Osx protein can be acetylated. Generally, acetylation inhibits DNA binding when the acetylation sites are located in the DNA binding domain, and if they are adjacent to the DNA binding domain, DNA binding is activated. A recent study has identified K307 and K312 as the acetylation sites of Osx (Lu et al., 2016). Among them, K307 is close to the C2H2 DNA binding domain of Osx (from amino acids 309 to 376), while K312 is located at the N-terminus of Osx’s DNA-binding domain. It is necessary to take further exploration for understanding of the structural changes of Osx after acetylation and the signal pathway networks about acetylation or deacetylation of Osx, as well as any crosstalk between acetylation and other post-translational modifications.

DNA methylation has been a hot topic of epigenetic studies in the bone development system. The CpG dinucleotides of the Osx promoter regions were unmethylated in osteogenic cell lines transcribing but methylated in non-osteogenic cell lines, suggesting that DNA methylation plays an important role in cell type-specific expression of Osx (Lee et al., 2006; Sepulveda et al., 2017a). DNA demethylation is accompanied by activation of the Osx gene during osteoblast differentiation, which involves the release of DNA methyltransferases from the Osx promoter (Sepulveda et al., 2017b).

## The Potential Efficacy of Osx for the Treatment of Osteolytic Diseases

In the normal physiological circumstances, bone resorption by osteoclasts and bone formation by osteoblasts maintain a healthy balance. Once the balance of this coupled process is broken, the molecular characteristics of the bone microenvironment change. As a result, osteolytic lesions occur, eliciting severe bone pain and fractures.

Osteoporosis is an osteolytic disease resulted from imbalance in bone homeostasis. It is well-known that estrogen (ER) therapy can significantly achieve anti-fracture efficacy, especially for postmenopausal women (Farr et al., 2019). Osx is also involved in osteoporosis treatment. However, ER therapy is usually accompanied by increased risk of breast, ovarian and endometrial cancer, which eventually leads to its diminished application clinically. In addition, as the only drug stimulating bone formation approved by Food and Drug Administration (FDA), Parathyroid hormone (PTH) has been proved to increase the Osx expression levels. However, it is related to osteosarcoma and can only be used for 2 years (Arumugam et al., 2019; Yang et al., 2019). Therefore, big efforts are underway to investigate new drugs or find serendipitous effects with old drugs in order to gain better therapy efficacy and minimize potential harms of long-term drug exposure (Stringhetta-Garcia et al., 2016; Qaseem et al., 2017; Haryati et al., 2018; Farr et al., 2019; Yoon et al., 2019). Besides, some natural extracts have been identified to promote osteogenic differentiation via Osx in the treatment of osteoporosis (Choi et al., 2016; Yashan et al., 2017; Wang et al., 2018; Chai et al., 2019; El-Makawy et al., 2020). They positively regulate the transcriptional expression and enhance the activity of Osx via different signaling pathways. Recent research has illustrated that the initial anabolic response after mechanical loading is based on the activation and proliferation of Osx lineage cells, but not the differentiation of progenitor cells (Zannit and Silva, 2019). Low intensity pulsed ultrasound (LIPUS) has been proven successful recoveries from non-unions, delayed unions and fracture of the bone in both animal experiments and clinical treatments. The underlying mechanism revealed that LIPUS-mediated mechanism of osteogenic differentiation may be achieved via upregulation of BMP2 expression and through activation of the BMP/Smad canonical pathway, and then increased Osx expression (Maung et al., 2020).

In addition to osteoporosis, Osx may be an attractive therapeutic target for the control of other osteolytic diseases such as osteosarcoma and bone metastasis of cancers. The expression of Osx was decreased in murine osteosarcoma cells compared with normal mouse osteoblasts. The transfection of Osx into K7M2 cells altered the osteolytic morphology of the tumors. More specifically, the expression of Osx suppressed the osteolytic phenotype (Cao et al., 2005). Decreased Osx expression would result in decreased osteoblast differentiation and increased osteoclast activity leading to lytic destruction as the tumor cells invade the normal bone. Multiple myeloma (MM) is a malignancy to involve the skeleton with patients developing osteolytic bone lesions. A proteasome inhibitor and immunomodulatory drug bortezomib has been introduced in the therapy of MM. when treatment with 10 nM of bortezomib, an increase of Osx RNA transcription both in normal and MM osteoblasts were observed. In the myeloma microenvironment, bortezomib has the ability to stimulate osteoblast differentiation by increasing Osx levels during osteogenesis and inhibit osteoclast differentiation by reducing the induction of osteoclast marker genes and proteins like NFATc1 (De Matteo et al., 2010; Kim et al., 2018; Wang H. et al., 2020). On the contrary, another proteasome inhibitor and immunomodulatory drug named lenalidomide induces osteoblast differentiation by inhibiting the secretion of osteoclastogenic factors which reflects the inhibitory effect exerted on osteogenic cells, but have no effect on Runx2 and Osx transcription (De Matteo et al., 2010). There is a strong nuclear expression of Osx in osteoid osteomas and osteoblastomas, while the expression of Osx in chondromyxoid fibromas and chondroblastomas are negative generally, which represent a novel marker in assessing chondroblastic and osteoblastic lineage differentiation of bone tumors (Dancer et al., 2010). The potential role of Osx in chondroblastoma are required to be explored indeeply.

Obviously, these studies suggest that Osx has become one of the few downstream specific transcription factors directly regulated by ER or other alternative drugs in the treatment of osteolytic diseases as shown in Table 2. And Osx might be a therapeutic target for osteosarcoma and other osteolytic diseases. Therefore, we would conclude that Osx-mediated mechanism of osteogenic differentiation points out the direction for exploitation of novel bone disease therapy strategies.

**TABLE 2:** The treatment for different osteolytic diseases by targeting Osx.

**Drug and trials**	**Disease or cells**	**Mechanisms**	**References**
Estrogen (ER)	Osteoporosis	By elevating the expressions of Runx2 and Osx	Han et al., 2020
Parathyroid hormone (PTH)	Dental pulp stem cells	By activating Erk and p38 signaling pathways and elevating Osx expression	Ge et al., 2020
Pseudoshikonin I	C2C12 cells	By stimulating Osx and Runx2 via the Akt and Pka signaling pathways	Choi et al., 2018
Gushukang (GSK)	Osteoporosis	By enhancing BMP2/Smads signaling pathway and elevating Osx expression	Chai et al., 2019
Strength training and Raloxifene	Osteopenia	By stimulating/reducing the genesis and activity of osteoblasts/osteoclasts	Stringhetta-Garcia et al., 2016
Turnip bioactive lipids	Osteoporosis	By activating Osx and suppressing Cathepsin K and TNF-α signaling	El-Makawy et al., 2020
Remifentanil	C2C12 cells	By upregulating Osx and Runx2 expression.	Yoon et al., 2019
Low intensity pulsed ultrasound(LIPUS)	Periosteum-derived cells	By upregulating Osx expression through activation of the BMP/Smad canonical pathway	Maung et al., 2020
Bortezomib	Multiple myeloma	By increasing Osx expression and synthesizing the final differentiation markers in osteogenesis	De Matteo et al., 2010
Lenalidomide	Multiple myeloma	By inhibiting the secretion of osteoclastogenic factors exerting on osteogenic cells negatively and no effect on Runx2 and Osx transcription	De Matteo et al., 2010

## Conclusion

Overall, the current literatures have demonstrated that Osx plays a critical role in osteogenesis differentiation. In the last 10 years, the mechanism of Osx in osteoblast differentiation and bone formation are further understood. The efforts in designing new drugs steadily increased due to the recognition that the significance of bone health. Noteworthy, Osx is involved in the complex communications among different bone cells and plays a role in the bone micro-environment. What’s more, Osx plays an important role in the treatment of osteolytic diseases. And Osx may be an attractive therapeutic target for the control of other osteolytic diseases. As more and more key genes and regulatory mechanisms of osteolytic diseases are discovered, medication of osteolytic diseases with new mechanisms is foreseeable in the future. We believe that the research on Osx and osteolytic diseases in the future will mainly focus on the following aspects: (1) The interaction between bone formation-related protein and Osx during osteogenic differentiation as well as their underlying molecular mechanisms remains to be further studied; (2) The new molecular mechanism of Osx regulated by miRNAs and lncRNAs remains to be explored, which may provide a potential target for the treatment of osteoporosis;(3) More effective and safer Osx-targeted drugs are needed to be further developed for the treatment of osteolytic diseases.

## Author Contributions

GW and YX conceived of this review. QL drafted this manuscript and designed the figures. ML conducted the literature investigation. All the authors provided the critical feedback, contributed to the discussion on the manuscript writing and revising, and approved the manuscript.

## Conflict of Interest

The authors declare that the research was conducted in the absence of any commercial or financial relationships that could be construed as a potential conflict of interest.

## References

[B1] Abdallah B. M. Alzahrani A. M. Kassem M. (2018). Secreted Clusterin protein inhibits osteoblast differentiation of bone marrow mesenchymal stem cells by suppressing ERK1/2 signaling pathway. *Bone* 110 221–229. 10.1016/j.bone.2018.02.018 29476977

[B2] Arumugam B. Vishal M. Shreya S. Malavika D. Rajpriya V. He Z. (2019). Parathyroid hormone-stimulation of Runx2 during osteoblast differentiation via the regulation of lnc-SUPT3H-1:16 (RUNX2-AS1:32) and miR-6797-5p. *Biochimie* 158 43–52. 10.1016/j.biochi.2018.12.006 30562548

[B3] Azetsu Y. Inohaya K. Takano Y. Kinoshita M. Tasaki M. Kudo A. (2017). The sp7 gene is required for maturation of osteoblast-lineage cells in medaka (*Oryzias latipes*) vertebral column development. *Dev. Biol.* 431 252–262. 10.1016/j.ydbio.2017.09.010 28899668

[B4] Baek W. Y. Kim J. E. (2011). Gene alterations by osteoblast-specific transcription factor osterix in postnatal bone formation. *Osteoporosis* 9 51–56.

[B5] Barbuto R. Mitchell J. (2013). Regulation of the osterix (Osx, Sp7) promoter by osterix and its inhibition by parathyroid hormone. *J. Mol. Endocrinol.* 51 99–108. 10.1530/jme-12-0251 23682129 PMC3685218

[B6] Calabrese G. M. Mesner L. D. Stains J. P. Tommasini S. M. Horowitz M. C. Rosen C. J. (2017). Integrating GWAS and co-expression network data identifies bone mineral density genes SPTBN1 and MARK3 and an osteoblast functional module. *Cell Syst.* 4 46–59.e4. 27866947 10.1016/j.cels.2016.10.014 PMC5269473

[B7] Canalis E. Schilling L. Eller T. Yu J. (2020). Nuclear factor of activated T cells 1 and 2 are required for vertebral homeostasis. *J. Cell. Physiol.* 235 8520–8532. 10.1002/jcp.29696 32329053 PMC7529842

[B8] Cao Y. Zhou Z. de Crombrugghe B. Nakashima K. Guan H. Duan X. (2005). Osterix, a transcription factor for osteoblast differentiation, mediates antitumor activity in murine osteosarcoma. *Cancer Res.* 65 1124–1128. 10.1158/0008-5472.Can-04-2128 15734992

[B9] Cao Z. Liu R. Zhang H. Liao H. Zhang Y. Hinton R. J. (2015). Osterix controls cementoblast differentiation through downregulation of Wnt-signaling via enhancing DKK1 expression. *Int. J. Biol. Sci.* 11 335–344. 10.7150/ijbs.10874 25678852 PMC4323373

[B10] Caparros-Martin J. Aglan M. Temtamy S. Martinez-Glez V. Valencia M. Tenorio J. (2013). “OSX/SP7 mutations and osteogenesis imperfecta,” in *Osteogenesis Imperfecta: A Translational Approach to Brittle Bone Disease*, eds Shapiro J. R. Byers P. H. Glorieux F. H. Sponsellor P. (Amsterdam: Elsevier), 173–179. 10.1016/B978-0-12-397165-4.00018-6

[B11] Chai S. Wan L. Wang J. L. Huang J. C. Huang H. X. (2019). Gushukang inhibits osteocyte apoptosis and enhances BMP-2/Smads signaling pathway in ovariectomized rats. *Phytomedicine* 64:153063. 10.1016/j.phymed.2019.153063 31419728

[B12] Chen D. Tian W. Li Y. Tang W. Zhang C. (2012). Osteoblast-specific transcription factor Osterix (Osx) and HIF-1α cooperatively regulate gene expression of vascular endothelial growth factor (VEGF). *Biochem. Biophys. Res. Commun.* 424 176–181. 10.1016/j.bbrc.2012.06.104 22750006

[B13] Chen X. Wang Z. Duan N. Zhu G. Schwarz E. M. Xie C. (2018). Osteoblast-osteoclast interactions. *Connect. Tissue Res.* 59 99–107. 10.1080/03008207.2017.1290085 28324674 PMC5612831

[B14] Chen Y. Chen M. Xue T. Li G. Wang D. Shang P. (2019). Osteocytic connexin 43 channels affect fracture healing. *J. Cell. Physiol.* 234 19824–19832. 10.1002/jcp.28581 30980397 PMC6660377

[B15] Choi Y. H. Gu Y. M. Oh J. W. Lee K. Y. (2011a). Osterix is regulated by Erk1/2 during osteoblast differentiation. *Biochem. Biophys. Res. Commun.* 415 472–478. 10.1016/j.bbrc.2011.10.097 22056560

[B16] Choi Y. H. Han Y. Jin S. W. Lee G. H. Kim G. S. Lee D. Y. (2018). Pseudoshikonin I enhances osteoblast differentiation by stimulating Runx2 and Osterix. *J. Cell. Biochem.* 119 748–757. 10.1002/jcb.26238 28657691

[B17] Choi Y. H. Han Y. Lee S. H. Jin Y. H. Bahn M. Hur K. C. (2015). Cbl-b and c-Cbl negatively regulate osteoblast differentiation by enhancing ubiquitination and degradation of Osterix. *Bone* 75 201–209. 10.1016/j.bone.2015.02.026 25744063

[B18] Choi Y. H. Jeong H. M. Jin Y.-H. Li H. Yeo C.-Y. Lee K.-Y. (2011b). Akt phosphorylates and regulates the osteogenic activity of Osterix. *Biochem. Biophys. Res. Commun.* 411 637–641. 10.1016/j.bbrc.2011.07.009 21777568

[B19] Choi Y. H. Kim G. S. Choi J. H. Jin S. W. Kim H. G. Han Y. (2016). Ethanol extract of *Lithospermum erythrorhizon* Sieb. et Zucc. promotes osteoblastogenesis through the regulation of Runx2 and Osterix. *Int. J. Mol. Med.* 38 610–618. 10.3892/ijmm.2016.2655 27353217

[B20] Dancer J. Y. Henry S. P. Bondaruk J. Lee S. Ayala A. G. de Crombrugghe B. (2010). Expression of master regulatory genes controlling skeletal development in benign cartilage and bone forming tumors. *Hum. Pathol.* 41 1788–1793. 10.1016/j.humpath.2010.06.008 21078438 PMC4012830

[B21] De Matteo M. Brunetti A. E. Maiorano E. Cafforio P. Dammacco F. Silvestris F. (2010). Constitutive down-regulation of Osterix in osteoblasts from myeloma patients: in vitro effect of Bortezomib and Lenalidomide. *Leuk. Res.* 34 243–249. 10.1016/j.leukres.2009.07.017 19656567

[B22] El-Makawy A. I. Ibrahim F. M. Mabrouk D. M. Abdel-Aziem S. H. Sharaf H. A. Ramadan M. F. (2020). Efficiency of turnip bioactive lipids in treating osteoporosis through activation of Osterix and suppression of Cathepsin K and TNF-alpha signaling in rats. *Environ. Sci. Pollut. Res. Int.* 27 20950–20961. 10.1007/s11356-020-08540-7 32253695

[B23] Farr J. N. Rowsey J. L. Eckhardt B. A. Thicke B. S. Fraser D. G. Tchkonia T. (2019). Independent roles of estrogen deficiency and cellular senescence in the pathogenesis of osteoporosis: evidence in young adult mice and older humans. *J. Bone Miner. Res.* 34 1407–1418. 10.1002/jbmr.3729 30913313 PMC6697189

[B24] Felber K. Elks P. M. Lecca M. Roehl H. H. (2015). Expression of osterix is regulated by FGF and Wnt/beta-catenin signalling during Osteoblast differentiation. *PLoS One* 10:e0144982. 10.1371/journal.pone.0144982 26689368 PMC4686927

[B25] Fu H. Doll B. Mcnelis T. Hollinger J. O. (2010). Osteoblast differentiation in vitro and in vivo promoted by Osterix. *J. Biomed. Mater. Res. A* 83A 770–778. 10.1002/jbm.a.31356 17559111

[B26] Fu X. Li Y. Huang T. Yu Z. Ma K. Yang M. (2018). Runx2/Osterix and zinc uptake synergize to orchestrate osteogenic differentiation and citrate containing bone apatite formation. *Adv. Sci.* 5:1700755. 10.1002/advs.201700755 29721422 PMC5908346

[B27] Gamez B. Rodriguez-Carballo E. Bartrons R. Rosa J. L. Ventura F. (2013). MicroRNA-322 (miR-322) and its target protein Tob2 modulate Osterix (Osx) mRNA stability. *J. Biol. Chem.* 288 14264–14275. 10.1074/jbc.M112.432104 23564456 PMC3656283

[B28] Gao M. Su Q. Liang T. Ma J. Zou X. (2018). Transcriptional activation of ENPP1 by osterix in osteoblasts and osteocytes. *Eur. Cell. Mater.* 36 1–14. 10.22203/ecm.v036a01 30047979

[B29] Gao Y. Jheon A. Nourkeyhani H. Kobayashi H. Ganss B. (2004). Molecular cloning, structure, expression, and chromosomal localization of the human Osterix (SP7) gene. *Gene* 341 101–110. 10.1016/j.gene.2004.05.026 15474293

[B30] Gao Y. Xiao F. Wang C. Wang C. Cui P. Zhang X. (2018). Long noncoding RNA MALAT1 promotes osterix expression to regulate osteogenic differentiation by targeting miRNA-143 in human bone marrow-derived mesenchymal stem cells. *J. Cell. Biochem.* 119 6986–6996. 10.1002/jcb.26907 29741283

[B31] Ge X. Li Z. Jing S. Wang Y. Li N. Lu J. (2020). Parathyroid hormone enhances the osteo/odontogenic differentiation of dental pulp stem cells via ERK and P38 MAPK pathways. *J. Cell. Physiol.* 235 1209–1221. 10.1002/jcp.29034 31276209

[B32] Han K. Wang F. Yu M. Xu B. (2020). Estrogen promotes osteogenic differentiation of bone marrow stem cells in patients with postmenopausal osteoporosis by elevating the expressions of Runx-2 and Osterix. *Panminerva Med.* 10.23736/s0031-0808.19.03791-1 [Epub ahead of print]. 31985184

[B33] Han Y. Cho D. H. Chung D. J. Lee K. Y. (2016a). Osterix plays a critical role in BMP4-induced promoter activity of connexin43. *Biochem. Biophys. Res. Commun.* 478 683–688. 10.1016/j.bbrc.2016.08.007 27498006

[B34] Han Y. Kim C. Y. Cheong H. Lee K. Y. (2016b). Osterix represses adipogenesis by negatively regulating PPARgamma transcriptional activity. *Sci. Rep.* 6:35655. 10.1038/srep35655 27752121 PMC5067693

[B35] Han Y. Kim Y. M. Kim H. S. Lee K. Y. (2017). Melatonin promotes osteoblast differentiation by regulating Osterix protein stability and expression. *Sci. Rep.* 7:5716. 10.1038/s41598-017-06304-x 28720849 PMC5515917

[B36] Haryati A. H. Jamia J. Nor A. Khairana H. Noor M. S. Norazlina M. (2018). Demethylbelamcandaquinone B (Dmcq B) is the active compound of *Marantodes pumilum* var. alata (Blume) Kuntze with osteoanabolic activities. *Molecules* 23:1686. 10.3390/molecules23071686 29997309 PMC6100564

[B37] Hayami T. Kapila Y. L. Kapila S. (2011). Divergent upstream osteogenic events contribute to the differential modulation of MG63 cell osteoblast differentiation by MMP-1 (collagenase-1) and MMP-13 (collagenase-3). *Matrix Biol.* 30 281–289. 10.1016/j.matbio.2011.04.003 21539914 PMC3116144

[B38] He P. Yu H. Jiang L. Chen Z. Wang S. Macrae V. E. (2020). Hdac9 inhibits medial artery calcification through down-regulation of Osterix. *Vasc. Pharmacol.* 132:106775 10.1016/j.vph.2020.106775 32702412

[B39] He S. Yang S. Zhang Y. Li X. Gao D. Zhong Y. (2019). LncRNA ODIR1 inhibits osteogenic differentiation of hUC-MSCs through the FBXO25/H2BK120ub/H3K4me3/OSX axis. *Cell Death Dis.* 10:947. 10.1038/s41419-019-2148-2 31827076 PMC6906393

[B40] Hojo H. Ohba S. He X. Lai L. P. McMahon A. P. (2016). Sp7/Osterix is restricted to bone-forming vertebrates where it acts as a Dlx co-factor in osteoblast specification. *Dev. Cell* 37 238–253. 10.1016/j.devcel.2016.04.002 27134141 PMC4964983

[B41] Huang C. Geng J. Wei X. Zhang R. Jiang S. (2016). MiR-144-3p regulates osteogenic differentiation and proliferation of murine mesenchymal stem cells by specifically targeting Smad4. *FEBS Lett.* 590 795–807. 10.1002/1873-3468.12112 26918315

[B42] Jiang Y. Wu W. Jiao G. Chen Y. Liu H. (2019). LncRNA SNHG1 modulates p38 MAPK pathway through Nedd4 and thus inhibits osteogenic differentiation of bone marrow mesenchymal stem cells. *Life Sci.* 228 208–214. 10.1016/j.lfs.2019.05.002 31055087

[B43] Jiao W. Zhang D. Wang D. Xu R. Tang L. Zhao M. (2019). MicroRNA-638 inhibits human aortic valve interstitial cell calcification by targeting Sp7. *J. Cell. Mol. Med.* 23 5292–5302. 10.1111/jcmm.14405 31140727 PMC6653209

[B44] Jing H. Su X. Gao B. Shuai Y. Chen J. Deng Z. (2018). Epigenetic inhibition of Wnt pathway suppresses osteogenic differentiation of BMSCs during osteoporosis. *Cell Death Dis.* 9:176. 10.1038/s41419-017-0231-0 29416009 PMC5833865

[B45] Karieb S. Fox S. W. (2012). Zinc modifies the effect of phyto-oestrogens on osteoblast and osteoclast differentiation in vitro. *Br. J. Nutr.* 108 1736–1745. 10.1017/S0007114511007355 22289672

[B46] Kawane T. Komori H. Liu W. Moriishi T. Miyazaki T. Mori M. (2014). Dlx5 and mef2 regulate a novel runx2 enhancer for osteoblast-specific expression. *J. Bone Miner. Res.* 29 1960–1969. 10.1002/jbmr.2240 24692107

[B47] Kemp J. P. Morris J. A. Medina-Gomez C. Forgetta V. Evans D. M. (2017). Identification of 153 new loci associated with heel bone mineral density and functional involvement of GPC6 in osteoporosis. *Nat. Genet.* 49 1468–1475. 10.1038/ng.3949 28869591 PMC5621629

[B48] Kim D. Ko Y. Park M. Kim B. Sohn H. Lim W. (2019). Regulation of osteosclerosis by inoculated Cd133(+) PC3 Cells in bone-marrow microenvironmental niches. *JBMR Plus* 3:e10189. 10.1002/jbm4.10189 31372592 PMC6659585

[B49] Kim S. H. Kim M. O. Kim H. J. Neupane S. Kim H. J. Lee J. H. (2018). Bortezomib prevents ovariectomy-induced osteoporosis in mice by inhibiting osteoclast differentiation. *J. Bone Miner. Metab.* 36 537–546. 10.1007/s00774-017-0871-2 29027021

[B50] Klein-Nulend J. Bakker A. D. Bacabac R. G. Vatsa A. Weinbaum S. (2013). Mechanosensation and transduction in osteocytes. *Bone* 54 182–190. 10.1016/j.bone.2012.10.013 23085083

[B51] Koga T. Matsui Y. Asagiri M. Kodama T. Takayanagi H. (2005). NFAT and Osterix cooperatively regulate bone formation. *Nat. Med.* 11 880–885. 10.1038/nm1270 16041384

[B52] Komori T. (2018). Runx2, an inducer of osteoblast and chondrocyte differentiation. *Histochem. Cell Biol.* 149 313–323. 10.1007/s00418-018-1640-6 29356961

[B53] Kusu N. Laurikkala J. Imanishi M. Usui H. Konishi M. Miyake A. (2003). Sclerostin is a novel secreted osteoclast-derived bone morphogenetic protein antagonist with unique ligand specificity. *J. Biol. Chem.* 278 24113–24117. 10.1074/jbc.M301716200 12702725

[B54] Landgraf P. Rusu M. Sheridan R. Sewer A. Iovino N. Aravin A. (2007). A mammalian microRNA expression atlas based on small RNA library sequencing. *Cell* 129 1401–1414. 10.1016/j.cell.2007.04.040 17604727 PMC2681231

[B55] Lee D. S. Choung H. W. Kim H. J. Gronostajski R. M. Yang Y. I. Ryoo H. M. (2014). NFI-C regulates osteoblast differentiation via control of osterix expression. *Stem Cells* 32 2467–2479. 10.1002/stem.1733 24801901

[B56] Lee D. S. Roh S. Y. Park J. C. (2018). The Nfic-osterix pathway regulates ameloblast differentiation and enamel formation. *Cell Tissue Res.* 374 531–540. 10.1007/s00441-018-2901-3 30091046

[B57] Lee H. Min S. Song Y. Park Y. H. Park J. B. (2019). Bone morphogenetic protein-7 upregulates genes associated with osteoblast differentiation, including collagen I, Sp7 and IBSP in gingiva-derived stem cells. *Exp. Ther. Med.* 18 2867–2876. 10.3892/etm.2019.7904 31555377 PMC6755424

[B58] Lee J. Y. Lee Y. M. Kim M. J. Choi J. Y. Park E. K. Kim S. Y. (2006). Methylation of the mouse DIx5 and Osx gene promoters regulates cell type-specific gene expression. *Mol. Cells* 22 182–188. 17085970

[B59] Lee M. H. Kwon T. G. Park H. S. Wozney J. M. Ryoo H. M. (2003). BMP-2-induced Osterix expression is mediated by Dlx5 but is independent of Runx2. *Biochem. Biophys. Res. Commun.* 309 689–694. 10.1016/j.bbrc.2003.08.058 12963046

[B60] Lee S. H. Jeong H. M. Han Y. Cheong H. Kang B. Y. Lee K. Y. (2015). Prolyl isomerase Pin1 regulates the osteogenic activity of Osterix. *Mol. Cell. Endocrinol.* 400 32–40. 10.1016/j.mce.2014.11.017 25463757

[B61] Lee S. J. Lee E. H. Park S. Y. Kim J. E. (2017). Induction of fibrillin-2 and periostin expression in Osterix-knockdown MC3T3-E1 cells. *Gene* 596 123–129. 10.1016/j.gene.2016.10.018 27751812

[B62] Lee Y. J. Park S. Y. Lee S. J. Boo Y. C. Choi J. Y. Kim J. E. (2015). Ucma, a direct transcriptional target of Runx2 and Osterix, promotes osteoblast differentiation and nodule formation. *Osteoarthr. Cartil.* 23 1421–1431. 10.1016/j.joca.2015.03.035 25865393

[B63] Li Y. Hu W. Han G. Lu W. Jia D. Hu M. (2018a). Involvement of bone morphogenetic protein-related pathways in the effect of aucubin on the promotion of osteoblast differentiation in MG63cells. *Chem. Biol. Interact.* 283 51–58. 10.1016/j.cbi.2018.02.005 29408431

[B64] Li Y. Jin D. Xie W. Wen L. Chen W. Xu J. (2018b). PPAR-γ and Wnt regulate the differentiation of MSCs into adipocytes and osteoblasts respectively. *Curr. Stem Cell Res. Ther.* 13 185–192. 10.2174/1574888x12666171012141908 29034841

[B65] Li Z. Wang W. Xu H. Ning Y. Fang W. Liao W. (2017). Effects of altered CXCL12/CXCR4 axis on BMP2/Smad/Runx2/Osterix axis and osteogenic gene expressions during osteogenic differentiation of MSCs. *Am. J. Transl. Res.* 9 1680–1693. 28469774 PMC5411917

[B66] Liu B. Wu S. Han L. Zhang C. (2015). beta-catenin signaling induces the osteoblastogenic differentiation of human pre-osteoblastic and bone marrow stromal cells mainly through the upregulation of osterix expression. *Int. J. Mol. Med.* 36 1572–1582. 10.3892/ijmm.2015.2382 26496941 PMC4678161

[B67] Liu L. He F. M. Li L. L. Hu J. A. (2004). [The expression of MMP-9, MMP-2 in the remodeling bone tissue around implant during unloaded period]. *Hua Xi Kou Qiang Yi Xue Za Zhi* 22 325–327. 15379320

[B68] Liu L. Zeng D. Chen Y. Zhou J. Liao Y. Shi B. (2020). Microarc oxidation surface of titanium implants promote osteogenic differentiation by activating ERK1/2-miR-1827-Osterix. *In Vitro Cell. Dev. Biol. Anim.* 56 296–306. 10.1007/s11626-020-00444-7 32270391

[B69] Lu J. Qu S. Yao B. Xu Y. Jin Y. Shi K. (2016). Osterix acetylation at K307 and K312 enhances its transcriptional activity and is required for osteoblast differentiation. *Oncotarget* 7 37471–37486. 10.18632/oncotarget.9650 27250035 PMC5122325

[B70] Machida M. Dubousset J. Yamada T. Kimura J. Saito M. Shiraishi T. (2006). Experimental scoliosis in melatonin-deficient C57BL/6J mice without pinealectomy. *J. Pineal Res.* 41 1–7. 10.1111/j.1600-079X.2005.00312.x 16842534

[B71] Matsubara T. Kida K. Yamaguchi A. Hata K. Ichida F. Meguro H. (2008). BMP2 regulates Osterix through Msx2 and Runx2 during osteoblast differentiation. *J. Biol. Chem.* 283 29119–29125. 10.1074/jbc.M801774200 18703512 PMC2662012

[B72] Maung W. M. Nakata H. Miura M. Miyasaka M. Kim Y. K. Kasugai S. (2020). Low intensity pulsed ultrasound stimulates osteogenic differentiation of periosteal cells in vitro. *Tissue Eng. Part A.* 10.1089/ten.TEA.2019.0331 [Epub ahead of print]. 32164486

[B73] Milona M. A. Gough J. E. Edgar A. J. (2003). Expression of alternatively spliced isoforms of human Sp7 in osteoblast-like cells. *BMC Genomics* 4:43. 10.1186/1471-2164-4-43 14604442 PMC280673

[B74] Moon Y. J. Yun C.-Y. Choi H. Kim J. R. Park B.-H. Cho E.-S. (2018). Osterix regulates corticalization for longitudinal bone growth via integrin β3 expression. *Exp. Mol. Med.* 50 1–11. 10.1038/s12276-018-0119-9 30022046 PMC6052162

[B75] Nakajima K. Kho D. H. Yanagawa T. Harazono Y. Gao X. Hogan V. (2014). Galectin-3 inhibits osteoblast differentiation through notch signaling. *Neoplasia* 16 939–949. 10.1016/j.neo.2014.09.005 25425968 PMC4240919

[B76] Nakashima K. Zhou X. Kunkel G. Zhang Z. Deng J. M. Behringer R. R. (2002). The novel zinc finger-containing transcription factor osterix is required for osteoblast differentiation and bone formation. *Cell* 108 17–29. 10.1016/s0092-8674(01)00622-5 11792318

[B77] Nakatani T. Chen T. Partridge N. C. (2016). MMP-13 is one of the critical mediators of the effect of HDAC4 deletion on the skeleton. *Bone* 90 142–151. 10.1016/j.bone.2016.06.010 27320207 PMC4970950

[B78] Nemoto E. Sakisaka Y. Tsuchiya M. Tamura M. Nakamura T. Kanaya S. (2016). Wnt3a signaling induces murine dental follicle cells to differentiate into cementoblastic/osteoblastic cells via an osterix-dependent pathway. *J. Periodontal. Res.* 51 164–174. 10.1111/jre.12294 26095156

[B79] Niger C. Lima F. Yoo D. J. Gupta R. R. Buo A. M. Hebert C. (2011). The transcriptional activity of osterix requires the recruitment of Sp1 to the osteocalcin proximal promoter. *Bone* 49 683–692. 10.1016/j.bone.2011.07.027 21820092 PMC3170016

[B80] Niu P. Zhong Z. Wang M. Huang G. Xu S. Hou Y. (2017). Zinc finger transcription factor Sp7/Osterix acts on bone formation and regulates col10a1a expression in zebrafish. *Sci. Bull.* 62 174–184. 10.1016/j.scib.2017.01.009 36659402

[B81] Oh J. H. Park S. Y. de Crombrugghe B. Kim J. E. (2012). Chondrocyte-specific ablation of Osterix leads to impaired endochondral ossification. *Biochem. Biophys. Res. Commun.* 418 634–640. 10.1016/j.bbrc.2012.01.064 22290230 PMC4012832

[B82] Omoteyama K. Takagi M. (2010). The effects of Sp7/Osterix gene silencing in the chondroprogenitor cell line, ATDC5. *Biochem. Biophys. Res. Commun.* 403 242–246. 10.1016/j.bbrc.2010.11.023 21075078

[B83] Onizuka S. Iwata T. Park S. J. Nakai K. Yamato M. Okano T. (2016). ZBTB16 as a downstream target gene of Osterix regulates osteoblastogenesis of human multipotent mesenchymal stromal cells. *J. Cell. Biochem.* 117 2423–2434. 10.1002/jcb.25634 27335174 PMC5094493

[B84] Ortuño M. J. Ruiz-Gaspà S. Rodríguez-Carballo E. Susperregui A. R. Bartrons R. Rosa J. L. (2010). p38 regulates expression of osteoblast-specific genes by phosphorylation of Osterix. *J. Biol. Chem.* 285 31985–31994. 10.1074/jbc.M110.123612 20682789 PMC2952199

[B85] Ortuno M. J. Susperregui A. R. Artigas N. Rosa J. L. Ventura F. (2013). Osterix induces Col1a1 gene expression through binding to Sp1 sites in the bone enhancer and proximal promoter regions. *Bone* 52 548–556. 10.1016/j.bone.2012.11.007 23159876

[B86] Peng Y. Shi K. Wang L. Lu J. Li H. Pan S. (2013). Characterization of Osterix protein stability and physiological role in osteoblast differentiation. *PLoS One* 8:e56451. 10.1371/journal.pone.0056451 23457570 PMC3574093

[B87] Pérez-Campo F. M. Santurtún A. García-Ibarbia C. Pascual M. A. Valero C. Garcés C. (2016). Osterix and RUNX2 are transcriptional regulators of sclerostin in human bone. *Calcif. Tissue Int.* 99 302–309. 10.1007/s00223-016-0144-4 27154028

[B88] Pivetta E. Scapolan M. Pecolo M. Wassermann B. Abu-Rumeileh I. Balestreri L. (2011). MMP-13 stimulates osteoclast differentiation and activation in tumour breast bone metastases. *Breast Cancer Res.* 13:R105. 10.1186/bcr3047 22032644 PMC3262218

[B89] Qaseem A. Forciea M. A. McLean R. M. Denberg T. D. Clinical Guidelines Committee of the American College of Physicians (2017). Treatment of low bone density or osteoporosis to prevent fractures in men and women: a clinical practice guideline update from the American college of physicians. *Ann. Intern. Med.* 166 818–839. 10.7326/m15-1361 28492856

[B90] Qi J. Hu K. S. Yang H. L. (2015). Roles of TNF-α, GSK-3β and RANKL in the occurrence and development of diabetic osteoporosis. *Int. J. Clin. Exp. Pathol.* 8 11995–12004. 26722385 PMC4680330

[B91] Qu S. Wu J. Bao Q. Yao B. Duan R. Chen X. (2019). Osterix promotes the migration and angiogenesis of breast cancer by upregulation of S100A4 expression. *J. Cell. Mol. Med.* 23 1116–1127. 10.1111/jcmm.14012 30450809 PMC6349213

[B92] Rajamannan N. M. (2019). TIEG1 is upregulated in Lrp5/6-mediated valve osteogenesis. *J. Cell. Biochem.* 120 3362–3366. 10.1002/jcb.27606 30246479 PMC6336497

[B93] Ramazzotti G. Fiume R. Chiarini F. Campana G. Ratti S. Billi A. M. (2019). Phospholipase C-β1 interacts with cyclin E in adipose- derived stem cells osteogenic differentiation. *Adv. Biol. Regul.* 71 1–9. 10.1016/j.jbior.2018.11.001 30420274

[B94] Renn J. Winkler C. (2009). Osterix-mCherry transgenic medaka for in vivo imaging of bone formation. *Dev. Dyn.* 238 241–248. 10.1002/dvdy.21836 19097055

[B95] Rios-Arce N. D. Dagenais A. Feenstra D. Coughlin B. Kang H. J. Mohr S. (2020). Loss of interleukin-10 exacerbates early Type-1 diabetes-induced bone loss. *J. Cell. Physiol.* 235 2350–2365. 10.1002/jcp.29141 31538345 PMC6899206

[B96] Sakisaka Y. Kanaya S. Nakamura T. Tamura M. Shimauchi H. Nemoto E. (2016). p38 MAP kinase is required for Wnt3a-mediated osterix expression independently of Wnt-LRP5/6-GSK3β signaling axis in dental follicle cells. *Biochem. Biophys. Res. Commun.* 478 527–532. 10.1016/j.bbrc.2016.07.076 27450807

[B97] Sepulveda H. Aguilar R. Prieto C. P. Bustos F. Aedo S. Lattus J. (2017a). Epigenetic signatures at the RUNX2-P1 and Sp7 gene promoters control osteogenic lineage commitment of umbilical cord-derived mesenchymal stem cells. *J. Cell. Physiol.* 232 2519–2527. 10.1002/jcp.25627 27689934

[B98] Sepulveda H. Villagra A. Montecino M. (2017b). Tet-mediated DNA demethylation is required for SWI/SNF-dependent chromatin remodeling and histone-modifying activities that trigger expression of the Sp7 osteoblast master gene during mesenchymal lineage commitment. *Mol. Cell. Biol.* 37:e00177–17. 10.1128/MCB.00177-17 28784721 PMC5615189

[B99] Shi K. Lu J. Zhao Y. Wang L. Li J. Qi B. (2013). MicroRNA-214 suppresses osteogenic differentiation of C2C12 myoblast cells by targeting Osterix. *Bone* 55 487–494. 10.1016/j.bone.2013.04.002 23579289

[B100] Shi L. Cai G. Shi J. Guo Y. Chen D. Chen D. (2016). Ossification of the posterior ligament is mediated by osterix via inhibition of the β-catenin signaling pathway. *Exp. Cell Res.* 349 53–59. 10.1016/j.yexcr.2016.09.019 27693496

[B101] Sinha K. M. Yasuda H. Coombes M. M. Dent S. Y. de Crombrugghe B. (2010). Regulation of the osteoblast-specific transcription factor Osterix by NO66, a Jumonji family histone demethylase. *EMBO J.* 29 68–79. 10.1038/emboj.2009.332 19927124 PMC2780536

[B102] Sinha K. M. Yasuda H. Zhou X. de Crombrugghe B. (2014). Osterix and NO66 histone demethylase control the chromatin of Osterix target genes during osteoblast differentiation. *J. Bone Miner. Res.* 29 855–865. 10.1002/jbmr.2103 24115157 PMC3961497

[B103] Stringhetta-Garcia C. T. Singulani M. P. Santos L. F. Louzada M. J. Nakamune A. C. Chaves-Neto A. H. (2016). The effects of strength training and raloxifene on bone health in aging ovariectomized rats. *Bone* 85 45–54. 10.1016/j.bone.2015.11.023 26812611

[B104] Subramaniam M. Pitel K. S. Withers S. G. Drissi H. Hawse J. R. (2016). TIEG1 enhances Osterix expression and mediates its induction by TGFbeta and BMP2 in osteoblasts. *Biochem. Biophys. Res. Commun.* 470 528–533. 10.1016/j.bbrc.2016.01.112 26801561 PMC4747784

[B105] Sun C. Yuan H. Wang L. Wei X. Williams L. Krebsbach P. H. (2016). FAK promotes Osteoblast progenitor cell proliferation and differentiation by enhancing Wnt signaling. *J. Bone Miner. Res.* 31 2227–2238. 10.1002/jbmr.2908 27391080 PMC5642940

[B106] Sun X. Yang X. Zhao Y. Li Y. Guo L. (2018). Effects of 17beta-estradiol on mitophagy in the murine MC3T3-E1 osteoblast cell line is mediated via G protein-coupled estrogen receptor and the ERK1/2 signaling pathway. *Med. Sci. Monit.* 24 903–911. 10.12659/msm.908705 29438359 PMC5819311

[B107] Sun Y. Cai M. Zhong J. Yang L. Xiao J. Jin F. (2019). The long noncoding RNA lnc-ob1 facilitates bone formation by upregulating Osterix in osteoblasts. *Nat. Metab.* 1 485–496. 10.1038/s42255-019-0053-8 32694877

[B108] Suske G. Bruford E. Philipsen S. (2005). Mammalian SP/KLF transcription factors: bring in the family. *Genomics* 85 551–556. 10.1016/j.ygeno.2005.01.005 15820306

[B109] Takarada T. Nakazato R. Tsuchikane A. Fujikawa K. Iezaki T. Yoneda Y. (2016). Genetic analysis of Runx2 function during intramembranous ossification. *Development* 143 211–218. 10.1242/dev.128793 26657773

[B110] Tang W. Yang F. Li Y. de Crombrugghe B. Jiao H. Xiao G. (2012). Transcriptional regulation of vascular endothelial growth factor (VEGF) by osteoblast-specific transcription factor Osterix (Osx) in osteoblasts. *J. Biol. Chem.* 287 1671–1678. 10.1074/jbc.M111.288472 22110141 PMC3265850

[B111] Wang C. G. Liao Z. Xiao H. Liu H. Hu Y. H. Liao Q. D. (2019). LncRNA KCNQ1OT1 promoted BMP2 expression to regulate osteogenic differentiation by sponging miRNA-214. *Exp. Mol. Pathol.* 107 77–84. 10.1016/j.yexmp.2019.01.012 30703347

[B112] Wang H. Cui Y. Luan J. Zhou X. Li C. Li H. (2017). MiR-5100 promotes osteogenic differentiation by targeting Tob2. *J. Bone Miner. Metab.* 35 608–615. 10.1007/s00774-016-0799-y 27873073

[B113] Wang H. Zhang H. Srinivasan V. Tao J. Sun W. Lin X. (2020). Targeting Bortezomib to bone increases its bone anabolic activity and reduces systemic adverse effects in mice. *J. Bone Miner. Res.* 35 343–356. 10.1002/jbmr.3889 31610066 PMC10587833

[B114] Wang Q. Zhao Y. Sha N. Zhang Y. Li C. Zhang H. (2018). The systemic bone protective effects of Gushukang granules in ovariectomized mice by inhibiting osteoclastogenesis and stimulating osteoblastogenesis. *J. Pharmacol. Sci.* 136 155–164. 10.1016/j.jphs.2018.01.007 29501580

[B115] Wang Z. Liu Q. Liu C. Tan W. Tang M. Zhou X. (2020). Mg(2+) in beta-TCP/Mg-Zn composite enhances the differentiation of human bone marrow stromal cells into osteoblasts through MAPK-regulated Runx2/Osx. *J. Cell. Physiol.* 235 5182–5191. 10.1002/jcp.29395 31742679

[B116] Wu J. Zhao J. Sun L. Pan Y. Wang H. Zhang W. B. (2018). Long non-coding RNA H19 mediates mechanical tension-induced osteogenesis of bone marrow mesenchymal stem cells via FAK by sponging miR-138. *Bone* 108 62–70. 10.1016/j.bone.2017.12.013 29253550

[B117] Wu L. Wu Y. Lin Y. Jing W. Nie X. Qiao J. (2007). Osteogenic differentiation of adipose derived stem cells promoted by overexpression of osterix. *Mol. Cell. Biochem.* 301 83–92. 10.1007/s11010-006-9399-9 17206379

[B118] Wu R. Ruan J. Sun Y. Liu M. Sha Z. Fan C. (2018). Long non-coding RNA HIF1A-AS2 facilitates adipose-derived stem cells (ASCs) osteogenic differentiation through miR-665/IL6 axis via PI3K/Akt signaling pathway. *Stem Cell Res. Ther.* 9:48. 10.1186/s13287-018-1082-z 30545407 PMC6293597

[B119] Wu Y. F. Matsuo N. Sumiyoshi H. Yoshioka H. (2010a). Sp7/Osterix is involved in the up-regulation of the mouse pro-α1(V) collagen gene (Col5a1) in osteoblastic cells. *Matrix Biol.* 29 701–706. 10.1016/j.matbio.2010.09.002 20888414

[B120] Wu Y. F. Matsuo N. Sumiyoshi H. Yoshioka H. (2010b). Sp7/Osterix up-regulates the mouse pro-alpha3(V) collagen gene (Col5a3) during the osteoblast differentiation. *Biochem. Biophys. Res. Commun.* 394 503–508. 10.1016/j.bbrc.2010.02.171 20206127

[B121] Xiao W.-l. Zhang D.-z. Fan C.-h. Yu B.-j. (2015). Intermittent stretching and osteogenic differentiation of bone marrow derived mesenchymal stem cells via the p38MAPK-osterix signaling pathway. *Cell. Physiol. Biochem.* 36 1015–1025. 10.1159/000430275 26112248

[B122] Xiao Z. S. Hjelmeland A. B. Quarles L. D. (2004). Selective deficiency of the “Bone-related” Runx2-II unexpectedly preserves osteoblast-mediated skeletogenesis. *J. Biol. Chem.* 279 20307–20313. 10.1074/jbc.m401109200 15007057

[B123] Xie J. Gu J. (2015). Identification of C-terminal Hsp70-interacting protein as a mediator of tumour necrosis factor action in osteoblast differentiation by targeting osterix for degradation. *J. Cell. Mol. Med.* 19 1814–1824. 10.1111/jcmm.12553 25818514 PMC4549032

[B124] Xing W. Godwin C. Pourteymoor S. Mohan S. (2019). Conditional disruption of the osterix gene in chondrocytes during early postnatal growth impairs secondary ossification in the mouse tibial epiphysis. *Bone Res.* 7:24. 10.1038/s41413-019-0064-9 31646014 PMC6804621

[B125] Xu Y. Yao B. Shi K. Lu J. Jin Y. Qi B. (2015). Phosphorylation of Serine422 increases the stability and transactivation activities of human Osterix. *FEBS Lett.* 589 857–864. 10.1016/j.febslet.2015.02.021 25728276

[B126] Yang B. Li S. Chen Z. Feng F. He L. Liu B. (2020). Amyloid β peptide promotes bone formation by regulating Wnt/β-catenin signaling and the OPG/RANKL/RANK system. *FASEB J.* 34 3583–3593. 10.1096/fj.201901550R 31944393

[B127] Yang F. Tang W. So S. de Crombrugghe B. Zhang C. (2010). Sclerostin is a direct target of osteoblast-specific transcription factor osterix. *Biochem. Biophys. Res. Commun.* 400 684–688. 10.1016/j.bbrc.2010.08.128 20816666 PMC4041335

[B128] Yang L. Cheng P. Chen C. He H.-B. Xie G.-Q. Zhou H.-D. (2012). miR-93/Sp7 function loop mediates osteoblast mineralization. *J. Bone Miner. Res.* 27 1598–1606. 10.1002/jbmr.1621 22467200

[B129] Yang M. Arai A. Udagawa N. Zhao L. Nishida D. Murakami K. (2019). Parathyroid hormone shifts cell fate of a leptin receptor-marked stromal population from adipogenic to osteoblastic lineage. *J. Bone Miner. Res.* 34 1952–1963. 10.1002/jbmr.3811 31173642

[B130] Yang Y. Huang Y. Zhang L. Zhang C. (2016). Transcriptional regulation of bone sialoprotein gene expression by Osx. *Biochem. Biophys. Res. Commun.* 476 574–579. 10.1016/j.bbrc.2016.05.164 27261434

[B131] Yano H. Hamanaka R. Nakamura-Ota M. Adachi S. Zhang J. J. Matsuo N. (2014). Sp7/Osterix induces the mouse pro-α2(I) collagen gene (Col1a2) expression via the proximal promoter in osteoblastic cells. *Biochem. Biophys. Res. Commun.* 452 531–536. 10.1016/j.bbrc.2014.08.100 25172663

[B132] Yao B. Wang J. Qu S. Liu Y. Jin Y. Lu J. (2019). Upregulated osterix promotes invasion and bone metastasis and predicts for a poor prognosis in breast cancer. *Cell Death Dis.* 10:28. 10.1038/s41419-018-1269-3 30631043 PMC6328543

[B133] Yashan Y. Yunxia L. Hong Z. (2017). Clinical study on the effects of Gushukang capsules in treating glucocorticoid induced osteoporosis. *Chin. J. Osteoporos.* 23 795–799.

[B134] Yoon J. Y. Kim T. S. Ahn J. H. Yoon J. U. Kim H. J. Kim E. J. (2019). Remifentanil promotes osteoblastogenesis by upregulating Runx2/osterix expression in preosteoblastic C2C12 cells. *J. Dent. Anesth. Pain Med.* 19 91–99. 10.17245/jdapm.2019.19.2.91 31065591 PMC6502765

[B135] You H. C. Jeong H. M. Jin Y. H. Li H. Yeo C. Y. Lee K. Y. (2011). Akt phosphorylates and regulates the osteogenic activity of Osterix. *Biochem. Biophys. Res. Commun.* 411 637–641. 10.1016/j.bbrc.2011.07.009 21777568

[B136] Yu T. Graf M. Renn J. Schartl M. Larionova D. Huysseune A. (2017). A vertebrate-specific and essential role forosterixin osteogenesis revealed by gene knockout in the teleost medaka. *Development* 144 265–271. 10.1242/dev.139550 27993982

[B137] Zannit H. M. Silva M. J. (2019). Proliferation and activation of osterix-lineage cells contribute to loading-induced periosteal bone formation in mice. *JBMR Plus* 3:e10227. 10.1002/jbm4.10227 31768488 PMC6874181

[B138] Zhang B. Zhang X. Xiao J. Zhou X. Chen Y. Gao C. (2020). Neuropeptide Y upregulates Runx2 and osterix and enhances osteogenesis in mouse MC3T3E1 cells via an autocrine mechanism. *Mol. Med. Rep.* 22 4376–4382. 10.3892/mmr.2020.11506 33000198 PMC7533442

[B139] Zhang C. Tang W. Li Y. (2012). Matrix metalloproteinase 13 (MMP13) is a direct target of osteoblast-specific transcription factor osterix (Osx) in osteoblasts. *PLoS One* 7:e50525. 10.1371/journal.pone.0050525 23185634 PMC3503972

[B140] Zhang H. Jiang Y. Qin C. Liu Y. Ho S. P. Feng J. Q. (2015). Essential role of osterix for tooth root but not crown dentin formation. *J. Bone Miner. Res.* 30 742–746. 10.1002/jbmr.2391 25349111 PMC4617775

[B141] Zhang H. Liu L. Jiang C. Pan K. Deng J. Wan C. (2020). MMP9 protects against LPS-induced inflammation in osteoblasts. *Innate Immun.* 26 259–269. 10.1177/1753425919887236 31726909 PMC7251795

[B142] Zhang J. F. Fu W. He M. L. Wang H. Wang W. M. Yu S. C. (2011). MiR-637 maintains the balance between adipocytes and osteoblasts by directly targeting Osterix. *Mol. Biol. Cell* 22 3955–3961. 10.1091/mbc.e11-04-0356 21880893 PMC3204058

[B143] Zhang S. Y. Gao F. Peng C. G. Zheng C. J. Wu M. F. (2018). miR-485-5p promotes osteoporosis via targeting Osterix. *Eur. Rev. Med. Pharmacol. Sci.* 22 4792–4799. 10.26355/eurrev_201808_15613 30070309

[B144] Zhang Z. H. Jia X. Y. Fang J. Y. Chai H. Huang Q. She C. (2020). Reduction of SOST gene promotes bone formation through the Wnt/beta-catenin signalling pathway and compensates particle-induced osteolysis. *J. Cell. Mol. Med.* 24 4233–4244. 10.1111/jcmm.15084 32134561 PMC7171346

[B145] Zhou X. Zhang Z. Feng J. Q. Dusevich V. M. Sinha K. Zhang H. (2010). Multiple functions of Osterix are required for bone growth and homeostasis in postnatal mice. *Proc. Natl. Acad. Sci. U.S.A.* 107 12919–12924. 10.1073/pnas.0912855107 20615976 PMC2919908

[B146] Zhou Y. Wang C. Si J. Wang B. Zhang D. Ding D. (2020). Melatonin up-regulates bone marrow mesenchymal stem cells osteogenic action but suppresses their mediated osteoclastogenesis via MT(2) -inactivated NF-κB pathway. *Br. J. Pharmacol.* 177 2106–2122. 10.1111/bph.14972 31900938 PMC7161576

